# Retrograde endoscopic lithotripsy using the innovative nanosecond electropulse method

**DOI:** 10.1186/2193-1801-2-538

**Published:** 2013-10-17

**Authors:** Alexander Gudkov, Vacheslav Boshchenko, Alexander Petlin, Vladimir Afonin, Valery Diamant, Marat Lerner

**Affiliations:** Department of Urology, Siberian State Medical University, Lenin Avenue #4, HC SSMU, Tomsk, 634050 Russia; Department of Urology, Military Medical Hospital, Sibirskaya Street #83A, TMH, Tomsk, 634023 Russia; Department of Urology, Medical Hospital No. 2, Bela Kuna Street #3, MCH #2, Tomsk, 634062 Russia; Medline Ltd. Street 20, Industrial Zone, Katsrin, Israel; Institute of Strength Physics and Materials Science of the Siberian Branch of the Russian Academy of Sciences, Academichesky av. 8/2, Tomsk, 634055 Russia

**Keywords:** Urolithiasis, Endoscopic lithotripsy, Nephrolithiasis, Nanosecond electropulse lithotripsy

## Abstract

**Purpose:**

The purpose of this clinical study is to assess the safety and efficiency of a novel lithotripsy method for endoscopic treatment of urinary stones throughout the urinary tract via semi-rigid and flexible endoscopes. This new method is based on the transfer of nanosecond high voltage electric pulses to the stones through flexible probes of various sizes.

**Methods:**

The study involved 879 patients aged 19-88 with renal, ureter and bladder calculi. Gender distribution: 46.3% female and 53.7% male. The prospective single-arm study took place at three centers. The goal of the clinical study was to evaluate the safety and efficacy of a novel lithotripsy method. All treatments were performed retrograde transurethrally. A variety of probes were used for stone fragmentation at different locations. Auxiliary treatments and adverse events were recorded as per protocol. Statistical analysis was conducted using SPSS software.

**Results:**

Nanosecond electropulse lithotripsy (NEPL) was found to be technically feasible for all patients with stones located in the kidney, UPJ, ureter and bladder. It requires only a few dozen pulses to disintegrate stones while causing only minor stone migration. The overall stone-free rate in the study was 96%. The average time required for executing the entire procedure was 45±28 min. The overwhelming majority of intraoperative complications occurred due to endoscopic manipulation when using a rigid ureterorenoscope and not due to lithotripsy impact.

**Conclusions:**

NEPL is a new, efficient and safe method for urinary stone disintegration that can be used throughout the urinary tract using rigid and flexible endoscopes. Intraoperative complications of the NEPL procedure do not exceed the percentage of adverse effects observed in other lithotripsy methods. The main advantages of relatively low-cost NEPL are fast stone fragmentation requiring only a few dozen pulses to disintegrate stones, tissue safety and availability of highly flexible probes for treating stones in the lower pole through a flexible ureterorenoscope.

## Introduction

Impact lithotripters (pneumatic, electrokinetic and ultrasonic) are considered to be efficient and safe. However, their application is limited to rigid endoscopes and their use in the proximal ureter (Martov et al. 1998;Santa-Cruz et al. 1998) is restricted due to stone migration. Laser and electrohydraulic are efficient lithotripsy methods (Martov et al. 1998;Devarajan et al. 1998;Yang & Hong 1996) that can be also used via actively deflectable, flexible endoscopes in all locations in the urinary tract. Electrohydraulic/laser lithotripsy probes have small diameters of 1.9 Fr./1.3-1.5 Fr. that allow high irrigation flow through the endoscope working channel and that can be used in all sections of the ureter, pelvis and calyx (Devarajan et al. 1998;Yang & Hong 1996;Grasso & Bagley 1998;Marks & Teichman 2007;Elashry et al. 1996). However, electrohydraulic lithotripsy (EHL) has a higher complication rate because the high intensity electrohydraulic shock wave that is generated can cause tissue damage when used in close proximity to the urothelium. A ureteric perforation rate of 17.6% is reported for EHL (Martov et al. 1998). It was for this reason that EHL has been widely abandoned, especially for use in the ureter where a safe distance cannot be maintained. Laser lithotripsy is safer than EHL but still is more expensive and provides slower disintegration (Yang & Hong 1996;Grasso & Bagley 1998;Marks & Teichman 2007;Elashry et al. 1996). Frequent damage to flexible ureterorenoscopes due to laser fiber breakage inside the deflected section of the flexible endoscope is a big drawback of laser lithotripsy.

Thus, a demand still exists for a universal endoscopic lithotripsy method with high tissue safety and probe flexibility that does not reduce flexible endoscope deflection or cause flexible endoscope damage. A new method for direct contact endoscopic lithotripsy based on the transmission of nanosecond duration electric pulses directly to the stone has been developed (Chernenko et al. 2007). The new Nanosecond Electropulse Lithotripsy (NEPL) method complies with this requirement profile.

While NEPL appears to be similar to EHL (they both use a probe with two electrodes at the distal end to which electric pulses are applied), their characteristics are fundamentally different. In contrast with EHL, which disintegrates stones by generating a shock wave in liquid, NEPL operates at much higher voltage and an ultrafast discharge time of less than 50 ns in direct contact with the stone. Urinary stones are usually non-conducting, but when the voltage across the stone becomes too great – i.e., if the electrostatic field becomes too intense – the stone will begin to spontaneously conduct current. Furthermore, under such high voltage and fast discharge, the dielectric resistance in the stone is below that of the liquid medium so that the electrical breakdown occurs through the stone and not through the liquid. This phenomenon was discovered in the 1960s (Vorobyev et al. 1961). The conceptual basis for the electropulse method of material destruction is described in (Semkin et al. 1995;Shuloyakov et al. 1995) and is illustrated by experimental data showing its significant potential. Presentation of the physical basis of the electropulse method is given in (Semkin et al. 1995) where the physical principles of electrical breakdown of solid dielectrics are considered. The so-called dielectric breakdown through the stone leads to tensile thermomechanical stresses in the stone resulting in its fragmentation (Kurets et al. 2002).

**Figure 1:**
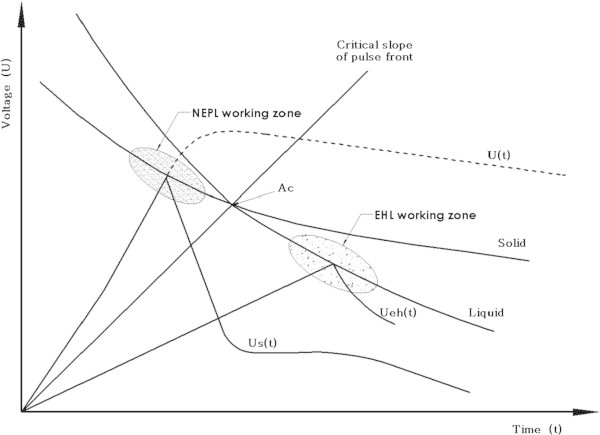
**The principle of NEPL; comparison of volt-second characteristics of solid state and of liquid media: Ac - the point where the probability of a breakdown through liquid or solid is equal; U(t) – pulse voltage in the absence of breakdown; Us(t) – pulse voltage under breakdown of a solid dielectric; Ueh(t) – pulse voltage during liquid breakdown.**

Figure 1 describes the voltage over time function when an electrical breakdown with the same discharge gap occurs in either a solid material or liquid medium. At the intersection point of the volt-second characteristics Ac, the probability that the electrical breakdown will occur through either the solid object or the liquid is equal. When exposed to a pulse voltage for less than 200-300 nanoseconds (to the left of point Ac), the dielectric strength in the solid object drops below the dielectric strength in the liquid so that the electrical breakdown occurs in the solid state (Semkin et al. 1995;Kurets et al. 2002).

Conventional EHL operates with a discharge time of several hundred microseconds at much lower voltage so that the electrical discharge and breakdown always occurs through the liquid (Figure 1), thus producing a shock wave in the liquid that can cause serious damage to nearby surrounding tissue.

In contrast, NEPL transfers the energy of the nanosecond electrical pulse discharges directly into the volume of the solid material by direct contact between the probe and the solid, where it creates a discharge plasma channel. A micro-explosion occurs during release of energy in the discharge channel in the solid resulting in formation of a crater and separation of a portion from the solid material. Microcracks caused by the electrical breakdown then begin to accumulate within the stone. After that, these microcracks combine and form a main crack connected with the initial pitted area between the electrodes and lead to the subsequent splitting of the stone (Semkin et al. 1995;Kurets et al. 2002;Martov et al. 2013). It should be noted that direct transfer of energy into the stone in NEPL makes this method effective and safe.

Tissue safety with the use of NEPL has thus been established. However, if the NEPL probe is located solely in a liquid and is not in direct contact with the stone, a discharge can occur through a liquid and produce a pressure wave when the pulse is released. In order to establish tissue safety in such a case, safety studies have been conducted on canines and on human ex-vivo tissue samples harvested after nephrectomy, ureterectomy and cystectomy procedures. In these studies, the probe tip was positioned close to or in direct contact with the tissue when the pulses were released. The low-trauma profile of NEPL has been demonstrated in-vivo on ureters and urinary bladders of sexually mature dogs (Boshchenko et al. 2012). A further study demonstrated that a direct nanosecond electropulse exposure of 1.0 J is safe for kidney, ureter and bladder urothelium (Gudkov et al. 2013).

The in-vitro efficiency of NEPL and Ho:YAG laser lithotripter was compared in (Martov et al. 2013) where it was shown that nanosecond electropulse lithotripsy is a more effective mode of stone disintegration and that their differing characteristics can be explained by essential differences in their mechanisms of stone destruction.

The NEPL studied in this work is currently licensed for clinical use by the Ministry of Health of the Russian Federation and is now being used by physicians in dozens of hospitals without the need for approval by ethics committees.

The purpose of this study, which is one of the first collective clinical studies of NEPL, is to analyze available data and to assess the safety and efficiency of this novel lithotripsy method.

## Materials and methods

879 patients eligible for treatment were enrolled in the study. Patient demographics were as follows: average age 51±27; age range 19–88; gender: 407 (46.3%) female and 472 (53.7%) male. All patients signed the informed consent form to participate in the study. 804 patients (91.5%) were enrolled following emergency hospitalization with renal colic and 75 patients (8.5%) were enrolled following a scheduled visit. Treatments were performed at one of the following hospitals located in Tomsk, Russia: State Medical University Hospital, Municipal Hospital No. 2 and Military Hospital.

The results were analyzed using standard biological and medical statistical methods and with use of SPSS software. A p-value ≤ 5% was chosen for evaluating the statistical significance of differences in the obtained results.

Table 1 shows the distribution of patients according to eligibility criteria under the study protocol.

**Table 1:** **Patient distribution according to eligibility criteria**

Entry criteria	Kidney/ UPJ (Group I, n = 54)	Ureter (Group II, n = 799)				Bladder (Group III, n = 26)
		Upper/3 (n = 164)	Middle/3 (n = 67)	Lower/3 (n = 568)	Ureter, total (n = 799)	
1^st^ criterion (stone < 6 mm, renal colic, disturbance of urodynamic, no tendency to discharge), n (%)	3/5	68	26	381	475	N/A
	(15%)	(42%)	(39%)	(67%)^⊕^	(60%)^⊕^	
2^nd^ criterion (stone ≥ 6 mm, renal colic, disturbance of urodynamic), n (%)	24/15	91	37	163	291	N/A
	(72%)^ΔΔ^	(55%)^ΔΔ^	(55%)	(29%)^Δ^	(36%)	
3^rd^ criterion (stone of any size, no renal colic, no urodynamic or kidney function disturbance), n (%)	4/3	5	2	17	24	26
	(13%)	(3%)^ΔΔΔ⊕^	(3%)	(3%)^ΔΔ^	(3%)	(100%)
4^th^ criterion (“Steinstrasse” following ESWL), n (%)	0	0	2	7	9	N/A
	(0%)	(0%)	(3%)	(1%)	(1%)	

Note: ⊕ - p < 0.05 - Significant differences as compared to UPJ concrements; Δ - p < 0.05; ΔΔ - p < 0.01; ΔΔΔ - p < 0.001 - Significant differences as compared to concrements in the middle third of the ureter.

Stone size was assessed radiographically (i.e. using X-ray computed tomography). Table 2 presents the distribution of stone size and location

**Table 2:** **Distribution of stone size and location**

Stone size (mm) and location	Kidney/UPJ (Group I n = 54)	Ureter (Group II, n = 799)				Bladder (Group III, n = 26)
		Upper/3 (n = 164)	Middle/3 (n = 67)	Lower/3 (n = 568)	Ureter total (n = 799)	
Up to 5, n (%)	6 (11%)	87 (53%)**	46 (69%)***^⊕⊕^	341 (60%)***^⊕^	474 (59%)***^⊕^	0 (0%)
5, to 10, n (%)	38 (70%)	56 (34%)	18 (27%)^⊕⊕^	201 (35%)	275 (34%)	3 (12%)
10, to 15, n (%)	6 (11%)	15 (9%)	3 (4%)	26 (5%)*	44 (6%)	9 (35%)
15, n (%)	4 (8%)	6 (4%)***	0 (0%)**	0 (0%)***	6 (1%)***	14 (53%)

Note: * - p < 0.05; ** - p < 0.01; *** - p < 0.001 – Significant differences as compared to urinary bladder concrements; ⊕ - p < 0.05; ⊕⊕ - p < 0.01 – Significant differences as compared to UPJ concrements.

The compatibility of stone type incidence in the study group with local prevalence data (See Table 3) confirms the random selection of patients (Polienko & Bakirov 2008).

**Table 3:** **Post treatment stone analysis**

Stone types according to stone analysis	Oxalate	Urate	Phosphate	Cystine
Relative incidence of stone types in study patients	48%	29%	22%	1%
Relative incidence of stone types according to available local prevalence data (Polienko & Bakirov 2008)	47%	30%	22%	1%

Patient pre-treatment examination and assessment included: symptoms/pain as reported by the patient; anamnesis; physical examination; urinalysis including bacteriological testing; clinical biochemistry; hematology; ultrasound examination of kidneys, ureters and bladder in B-mode; excretory urography for indications in cases of non-radiopaque; ureteral calculi – retrograde pneumopyeloureterography.

NEPL was carried out using the Urolith-105 M device (Lithotech Medical Ltd., Israel). The basic specifications are as follows: pulse front < 50 nanoseconds; pulse duration 250-500 nanoseconds; discharge voltage up to 10 kV; pulse energy range of 0.3 to 1.0 J. The high-voltage nanosecond pulse is transmitted to the stone through a special flexible coaxial cable in order to avoid transmission losses and signal distortion. Cable diameters vary in order to enable connection with the various French probe sizes. Coaxial cable is inserted into a polyimide sheath with which the probe’s flexibility can be controlled. A special tip is assembled on the probe’s distal end where the nanosecond discharge occurs when the probe is in contact with a stone.

All treatments were performed retrograde transurethrally with a cystoscope (bladder), semi-rigid ureteroscope (lower/mid ureter, and in some cases in the upper ureter) having a working channel of 5 ÷ 6 Fr (various manufacturers) or with a flexible ureterorenoscope (upper ureter, kidney) having a working channel of 3.6 Fr (Storz, ACMI). Various probe sizes were used for stone fragmentation: bladder – 4.5 Fr/650 mm long; mid-/lower ureter – 3.6 Fr/650-1,200 mm long; upper ureter and kidney via semi-rigid (3.6 Fr probe) or flexible (2.7 Fr probe) ureteroscope, 1,200 mm long. Single (various manufacturers) or a dual lumen (Flexor DL®, Cook Medical) ureteral access sheath was used systematically with a flexible URS.

Patients were treated either under general or spinal/peridural anesthesia.

Stone migration prevention or retrieval devices (N-Trap/N-Compass/N-Circle, Cook Medical Inc.) were used when clinically indicated and possible. It is important to note that no damage was caused to the migration prevention/retrieval devices by the NEPL device.

Direct contact between the lithotripsy probe and the stone was ensured by endoscopic visual monitoring. Pulse energy (0.5-1.0 J), frequency (one pulse mode or frequency mode) and number of pulses (quantity of pulses per pulse package) were set according to preliminarily evaluated stone density and size. Pulse release was controlled via a foot pedal. The probe was always in direct contact with the stone when starting a single pulse or pulse series. The probe tip was repositioned after each loss of stone contact or as determined by the physician. Lithotripsy was discontinued when fragment size was smaller than 1.5 mm as confirmed by direct endoscopic visualization or x-ray. At the end of the procedure, all patients routinely received a ureteral catheter or a stent on a case-by-case basis for 2-14 days. All patients underwent postoperative control observation one month following the procedure during routine visits to the clinic.

The stone-free criteria used in this study was a residual fragment size of ≤ 1.5 mm as confirmed by direct observation, direct postoperative imaging control and at the one month follow-up examination. Acceptance criteria for 'stone-free’ residual fragments after lithotripsy and stone retrieval varied in the range of ≤ 2 to 4 mm among the authors of the studies (Kiper et al. 2004;Teichman et al. 1997;Yeniyol et al. 2000;Macejko et al. 2009) and, in general, the stone-free criterion also depended largely on the experience of the operator (Yeniyol et al. 2000).

## Results

Table 4 presents the case groups according to energy/pulse rate settings used for the treatment and received stone free rate.

**Table 4:** **Lithotripter settings used to achieve successful fragmentation and complete fragmentation success rates**

NEPL parameters	Kidney/UPJ (Group I, n = 54)	Ureter (Group II, n = 799)				Bladder (Group III, n = 26)
		Upper/3 (n = 164)	Middle/3 (n = 67)	Lower/3 (n = 568)	Ureter, total (n = 799)	
Average energy per pulse, J	0.74±0.07	0.86±0.06	0.78±0.11	0.82±0.12	0.83±0.1	0.9±0.08
Pulse mode and pulse frequency, Hz	Single or double pulses,	Double pulses,	Double pulses,	Double pulses,	Double pulses,	Series of 3-5 pulses,
	5 Hz	5 Hz	5 Hz	5 Hz	5 Hz	5 Hz
Number of pulses required for concrement destruction, n	60±23*	61±48*	42±17*	29±15**	35±31**	136±57
% with complete fragmentation	Kidney: 96%	91%	100%	95%	n/a	100%
	UPJ 100%					

Note: * - p < 0.05; ** - p < 0.01 – Reliable differences as compared to urinary bladder concrements.

The average time for the entire procedure was 45±28 min. In 861 cases (98%), NEPL was followed by either extraction of the stone fragments using N-Compass (1.7 Fr or 2.4 Fr) or N-Circle (1.5 Fr or 2.2 Fr) baskets or by pulling fragments into the bladder using an N-Trap device.

Although NEPL energy pulse values in all of the groups are comparable, stone fragmentation in the pelvicalyceal system (PCS) and ureter required less average pulse energy than bladder stones (Table 4). It was found that the number of pulses (or total cumulative energy) required to fragment bladder stones was significantly higher than for kidney and ureter stones (136±57 vs. 60±23 in the kidneys and p < 0.05 and 136±57 vs. 35±31 in the ureters, p < 0.01) and is correlated with stone size (Tables 2 and 4). Single or double pulse mode at 5 Hz was used for stone fragmentation in the ureters and kidneys, while pulse series of 3-5 pulses at 5 Hz were used for bladder stones.

The overall stone-free rate in the study was 96%. 92.5% of patients become stone-free after a single session and 3.5% required a second session. The stone-free criteria was confirmed by direct observation, direct postoperative imaging control and at the one month follow-up examination.

Partial fragmentation was achieved in 4% of cases.

NEPL was technically feasible for all patients with stones located in the kidney, UPJ, ureter and bladder. Complete stone fragmentation in the kidney was achieved in 96% of cases and in the upper ureter in 91% of cases. In the UPJ and in the middle ureter, fragmentation success was 100%; stone destruction was successful in 95% of cases in the lower ureter (distal ureter part) and in 100% of cases for bladder stones.

Complications recorded during the study are presented in Table 5 (column 1, 2).

**Table 5:** **Complications and undesirable effects of retrograde ureteroscopy**

No.	Complications	Quantity	Notes
		(%)	
*I*	*Intraoperative*	63	Can be considered to a great extent as complications of ureteroscopy
		(7.4%)	
I-1	Ureter perforation	18	Related to mechanical impact, not electrical impact
		(2.1%)	
I-2	Migration of a concrement or its fragments to a kidney	45	
		(5.3%)	
*II*	*Macrohematuria*	140	To a great extent, due to the effect of endoscopic manipulation
		(16%)	
*III*	*Postoperative*		
III-1	Recurrence of renal colic (patients from Groups I and II).	88	Result of endoscopic manipulation
		(10.3%)	
III-2	Acute and chronic pyelonephritis, exacerbation of chronic cystitis	71	Result of endoscopic manipulation
		(8.1%)	
III-3	Acute retention of urine (Groups II and III)	6	Result of endoscopic manipulation
		(0.7%)	
*IV*	*Spontaneous discharge of fine concrement fragments*	194	Result of lithotripsy. Typical of any type of lithotripsy.
		(22%)	

## Discussion

The stone-free rate of 91% for NEPL on upper ureteric stones presented in this study is higher than or equal to the success rate reported for various laser endoscopic lithotripsy modalities as well as ESWL. Reported success rates in the upper ureter are in the range of 86% to 92% for laser lithotripsy and 55% to 61% for ESWL (Yang & Hong 1996;Eden et al. 1998;Bierkens et al. 1998;Lalak et al. 2002;Lam et al. 2002). It is therefore concluded that NEPL is an efficient method of stone treatment in the proximal ureter with efficiency close to that of laser lithotripsy. However, in the presence of high stone localization in the upper third of the ureter, the probability of concrement migration is higher during lithotripsy. Since it is not always possible to take action that would prevent stone migration with the use of various tools, the NEPL procedure was terminated in some stone migration cases, which accounts for the lower percentage of complete destruction of concrements located in the upper third of the ureter than in other cases.

The stone-free rate for NEPL in the distal ureter measured in the study is 95%. This is comparable to reported stone-free rates for other endoscopic contact lithotripsy modalities in that location (Eden et al. 1998;Bierkens et al. 1998) and considerably exceeds ESWL efficiency (Lalak et al. 2002). Thus NEPL is considered to be of equal or greater effectiveness in the treatment of distal and proximal ureteral calculus treatment than other modalities.

As for mid-ureter stones, NEPL achieved a 100% stone-free rate, which is comparable to laser lithotripsy (Bierkens et al. 1998;Sofer et al. 2002;Zhong et al. 2004).

This is true also with respect to the treatment of stones in the kidney, UPJ and bladder with NEPL success rates of 96%, 100% and 100% respectively.

Having demonstrated the comparable effectiveness of NEPL and laser lithotripsy, we then performed an indirect approximated comparison between these modalities in which the number of lithotripsy pulses required until stone fragmentation and operating time were measured. This evaluation was based on recently published laser lithotripsy studies and our own results using NEPL. According to the reviewed laser lithotripsy study (Lam et al. 2002), stone fragmentation was achieved in 20% of cases after 200–500 pulses, in 75% of cases after 500-1,500 pulses and in 5% of cases after >3,000 pulses. In our study, an average number of 60±23 pulses was required to achieve fragmentation of stones in the kidney and UPJ, 61±48 pulses for the upper third, 42±17 for the middle third and 29±15 for the lower third of the ureter. As for bladder stones, NEPL required an average of 136±57 pulses. These interim findings also correspond with results received in (Martov et al. 2013) where the efficacy of NEPL was compared in-vitro with the Ho:YAG laser lithotripter. These results show that, in general, fewer pulses (or cumulative energy and net time) are required for NEPL to achieve fragmentation of stones than for laser lithotripsy, often by several orders of magnitude.

However, NEPL efficiency would lack significance without clinical safety. In this regard, relevant data presented in Table 5 shows that 63 (7.4%) patients with kidney, UPJ and ureter calculi suffered from intraoperative complications, consisting of migration of a concrement or its fragments to a kidney (5.3%) or ureter perforation (2.1%). It should be noted that all cases of ureteral perforation as well as most cases of intraoperative complications during NEPL were observed in procedures where semi-rigid endoscopes were used. It should also be noted that all of the patients with ureter perforation had a complicated clinical course of urolithiasis (urethritis) with a stone larger than 8 mm present in the ureter for 6-9 days prior to NEPL that required extended disintegration time and manipulation of a semi-rigid ureteroscope in proximity to an edematous, loose wall of the ureter, which led to its perforation (see Notes in Table 5).

Numerous articles are devoted to complications that occur during retrograde semi-rigid ureteroscopy procedures (Tello Royloa et al. 1992;Geavlete et al. 2006;Abdelrahim et al. 2008;Taie et al. 2012). In the various articles, intraoperative complications were reported in 5.9%-28% of all procedures while ureteral perforations were observed in 0.65%-9% of cases. It was further stated that the number of adverse events during ureteroscopy and lithotripsy procedures is strongly dependent on the experience and qualifications of the physician. Therefore, the percentage of intraoperative complications that we recorded during this the multi-centered study correlates well with data in the literature for semi-rigid ureteroscopy. Thus, we believe that most cases of perforation were related to the mechanical force and not the electrical force applied by the urethroscope on the altered wall of the ureter. In 9 cases, the endoscopic intervention that had begun was terminated: six patients underwent open operation – ureterolithotomy – while in three other cases a stent was installed followed by successful repeated NEPL. In all other cases NEPL was successfully concluded.

Concrement migration was found to be more characteristic of patients with high concrement location. 29 patients with migrated concrements underwent complete operations with dynamic follow-up of the course of the disease. In the remaining 16 cases, the rigid ureteropyeloscope was replaced with a flexible ureteropyeloscope, which was inserted into the PCS, whereupon contact NEPL was performed in the pelvis or calyx.

Upon analysis of the cases of stone migration, we found that in some cases the stones migrated to the kidney before their exposure to the electrical pulses. Therefore, such cases should be regarded as adverse effects of retrograde ureteroscopy rather than complications of NEPL (see Notes in Table 5).

In our research, 140 (16%) patients had recorded episodes of macrohematuria on the day of the NEPL procedure, which in all cases stopped spontaneously within several hours without the need to prescribe hemostatic therapy. Our assessment is that this phenomenon is primarily a consequence of endoscopic manipulation rather than NEPL complication.

No intraoperative complications were recorded in the third group of patients.

Complications recorded during the early postoperative period are presented in Table 5, No. III. In our opinion, the above complications are the result of endoscopic manipulation rather than NEPL complication (see Notes in Table 5).

Spontaneous discharge of residual concrements of ≤1.5 mm during the postoperative period was recorded in 194 (22%) cases (Table 5). In 126 cases, the fragment discharge occurred spontaneously and required no additional intervention. In 68 (7.7%) cases, the discharge of fragments provoked relapse of renal colic and required ureteroscopic intervention with lithoextraction. No differences were found in the postoperative period between Groups I and II.

An early postoperative complication in Group III was exacerbation of chronic cystitis (4 cases (15%)) caused by both endoscopic manipulation and long-term exposure of the stone followed by inevitable injury to bladder mucosa, which occurs during any cystolithotripsy operation.

One (3.8%) patient from Group III experienced acute urine retention due to insertion of a large stone fragment into the neck of the urinary bladder. This required repeated ureteroscopy and NEPL of the stone fragment. Another patient (3.8%) from Group III experienced acute urine retention due to exacerbation of chronic prostatitis combined with benign hyperplasia of the prostate gland, which required the prescription of antibacterial and α1-adrenoceptor blocking agents.

No follow-up postoperative complications were recorded for one year of observation.

It is known that complications of intracorporeal lithotripsy include endoscopy-related conditions (potential injury to the urinary tract) and specific conditions related to incomplete stone fragmentation and incomplete fragment extraction that occasionally occur with all types of lithotripsy. The residual fragments can lead to renal or ureteral colic and repeated procedures. Even endoscopic lithoextraction leads to complications among which the most widely recognized are acute pyelonephritis observed according to (Bondarenko 2008) in 13.5% of patients and renal colic. According to (Hamid et al. 2005), complications of pneumatic lithotripsy can range from 5.35% to 12.6% for various post-treatment effects, while the complication rate for Ho:YAG laser ranges from 6% to 19% (Gettman & Segura 2007).

The average patient hospitalization time in the groups was in the range of 5.0±3.2 days, which is considerably shorter than after open lithotomy. 46% of urolithiasis patients were discharged from the hospital on the 3^rd^ day after NEPL and another 30% on the 5^th^ day.

## Conclusions

From the data of this single-arm study we can conclude that retrograde contact NEPL is thus an efficient and safe method of uroconcrement fragmentation that can break stones in all sections of the urinary tract. The average stone-free rate obtained in the study for NEPL is 96%. Most intraoperative complications observed in this work are not connected with the lithotripsy procedure. Adverse effects during the endoscopic manipulation and lithotripsy procedure in the work do not exceed the percentage of adverse effects shown in other lithotripsy methods. The main advantages of relatively inexpensive NEPL are: fast stone fragmentation, tissue safety and availability of highly flexible probes for treating stones in the lower pole through a flexible ureterorenoscope.
